# Contributions of naturalistic parent-child conversations to children’s science learning during informal learning at an aquarium and at home

**DOI:** 10.3389/fpsyg.2022.943648

**Published:** 2022-08-08

**Authors:** Grace Ocular, Kimberly R. Kelly, Lizbeth Millan, Savannah Neves, Kateri Avila, Betina Hsieh, Claudine Maloles

**Affiliations:** ^1^Child Language Interactions and Memory Lab, Department of Teacher Education, California State University, Long Beach, Long Beach, CA, United States; ^2^Child Language Interactions and Memory Lab, Department of Human Development, California State University, Long Beach, Long Beach, CA, United States; ^3^Department of Teacher Education, California State University, Long Beach, Long Beach, CA, United States

**Keywords:** informal STEM learning, parent elaboration, parent-child conversations, early childhood, funds of knowledge

## Abstract

This study examined the naturalistic conversations of 62 parent-child dyads during informal learning at an aquarium and with a subsample at home. Children (*M*_age_ = 69.8 months) with their parents were observed and audio recorded while exploring an aquarium exhibit, and a subset of dyads returned recorded home conversations reminiscing about the aquarium visit. Parent-child conversations at the aquarium were coded for child science talk and a range of parent talk variables, and parent-child conversations at home were coded for child science talk and talk about the value of the aquarium visit. Results revealed that parents tended to use more elaborative statements compared to other talk types in the aquarium. Yet, the different types of questions and statements that parents used with their children at the aquarium differentially related to their children’s science talk in the aquarium and while reminiscing at home. Findings highlight often-overlooked types of parent talk that provide meaningful ways for families to engage in science and may lead to positive child learning outcomes.

## Introduction

Young children meaningfully engage in science, technology, engineering, and mathematics (STEM) practices and begin to develop science habits of mind through early experiences (Campbell et al., 2018), which shape children’s science and educational outcomes (National Research Council, 2007; McClure et al., 2017). Informal learning environments (ILE) such as science museums and aquariums provide families with opportunities to engage with science in approachable ways. The home is also an important ILE in which families construct cultural knowledge about informal science processes and concepts (Riojas-Cortez et al., 2008). However, we know relatively little about how families connect their informal science learning between and across the informal spaces where children have early meaningful experiences. This issue is important because children and families may bring their *Funds of Knowledge (FoK)—*that is, historically undervalued skills and knowledge brought from home and community that are used during learning—(Moll et al., 1992; Basu and Calabrese Barton, 2007) to their participation in informal science across ILE spaces (Mathai, 2017), allowing everyday family practices in one space to build upon and strengthen the developing knowledge in another. This study examined parent-child conversations as families explored an aquarium exhibit, investigating to what extent parental talk connects with children’s talk about science at the aquarium and while reminiscing at home.

Aquariums are unique ILE. Visitor demographics and perceptions suggest they are comparatively more pluralistic spaces, with two-thirds of aquarium visitors earning less than $100,000 annually (Association of Zoos & Aquariums, 2021) compared to science museum visitors with incomes above $200,000 (Bingham, 2019). Visitor engagement with animals at aquariums fosters an educational experience that includes observation and information seeking (Kisiel et al., 2012), encouraging visitors to act as scientifically informed observers and participants (Cainey et al., 2012). Aquariums, as live animal settings, contribute to early development of biological knowledge (Geerdts et al., 2015) where children and parents have conversations that include talk about scientific processes, technology, math, and biological content (Kelly et al., 2020). Thus, aquariums promote learning about life sciences and conservationism (Ash et al., 2007; Bidart and Russell, 2017) and create opportunities for visitors to make connections with personal beliefs, attitudes, and experiences (Kisiel et al., 2012).

Parents and children also engage in informal science learning at home. Studies of home science literacy show that parents and children engage in science-related activities in various ways. Home science activity packets with prescribed prompts and questions engage families in scientific inquiry practices, including observation, recording data, and explanation (Strickler-Eppard et al., 2019). Parent-child dialogic science book reading with children at home supports children’s science knowledge gains (Mantzicopoulos et al., 2013). However, studies of science at home often focus on traditional science tasks and fewer have examined families’ everyday interactions. Cooking provides opportunities for parents and children to tap into their culture and existing knowledge about food and food preparation that can promote acquisition of science knowledge (e.g., measurement and chemical reactions) (Riojas-Cortez et al., 2008; Morris et al., 2021). Studies of family storytelling immediately following tinkering at a children’s science museum and days later at home (e.g., Jant et al., 2014; Marcus et al., 2021) also suggest the importance of investigating the families’ everyday science practices.

We take the view that children’s learning is a developmental process, and the development of thinking occurs within children’s everyday interactions (Vygotsky, 1978). From the sociocultural framework, children’s learning in community and home environments is situated within social interactions with expert others who guide children to attend to valued content and processes (Wood et al., 1976; Vygotsky, 1978). This view positions language as a potent cultural tool that mediates the acquisition of knowledge during learning activities and the development of new ways of thinking through dialog with others (Halliday, 1993; Schleppegrell, 2004). Within informal learning contexts, dialog between parents and children offers opportunities for engagement in scientific processes and science knowledge-building (Crowley et al., 2001a; Ash, 2003; Attisano et al., 2021).

Early family conversations are critical for the development of young children’s science thinking and learning (Callanan, 2012; Kelly et al., 2020) and may have long-term impacts on science interest and transfer of scientific knowledge to contexts beyond the ILE (Pagano et al., 2020). Conversations rich with elaborative language during informal learning allow children to use science-specific language (Marcus et al., 2017) and scaffold their short- and long-term science learning (Callanan and Jipson, 2001; Benjamin et al., 2010). Moreover, elaborative parent-child conversations at home following a science lesson at school boost children’s science learning (Leichtman et al., 2017).

From classic research, we know that parents who create highly elaborative contexts during day-to-day conversations with their children include more *wh*-questions and statements that add new information. Other parents use more yes/no questions, ask for the same information repetitively (Peterson and McCabe, 1992), creating a less elaborative context (Fivush et al., 2006). Parents who ask many *wh*-questions at science museums or as the event unfolds have children who show greater understanding, retention, and recall of their experiences during informal learning (Boland et al., 2003; Benjamin et al., 2010; Jant et al., 2014). Although the role of parent-child conversations in children’s science learning has been documented separately in the contexts of home and community learning spaces, less is known about how family conversations in one space extend, build upon, and exist within other learning spaces. To address this gap, we use a learning ecologies framework (Barron, 2006; Siemens, 2007) to explore and connect spaces where the development of informal science knowledge occurs. We view family reminiscing conversations as a tool that mediates family science engagement and children’s science learning across ILEs, allowing for the developmental gains in one space to transfer to another. Reminiscing conversations following an event influence the meaning that children make of the event, how memory is consolidated, and what information is retained and recalled (Ornstein et al., 2004; Haden, 2010) and are likely a natural extension of families’ at-home informal learning (Peterson and McCabe, 1994; Reese and Brown, 2000).

The current study examined family reminiscing after the ILE visit, because we view the content of such conversations as an outcome and continuation of family informal learning (Crowley et al., 2001a; Leinhardt et al., 2003). Moreover, family at-home conversations about their informal learning experiences may bridge children’s learning ecologies. Parent and child reflections contain science talk similar to that used during informal learning in a science museum (Marcus et al., 2017). Everyday family conversations can ultimately serve to bridge developmental processes occurring across learning ecologies. The *FoK* perspective (Moll et al., 1992) provides a counternarrative to deficit models of learning. From an *FoK* lens, we can look beyond the historically privileged ways of doing science to acknowledge science learning in everyday interactions. Reflecting on the value of the ILE visit may be one way that parents bridge children’s learning ecologies. Families may reflect on the value of informal learning experiences while exiting a museum exhibit (Pagano et al., 2019). However, research has not yet explored how families talk about the value of ILE visits when reminiscing at home.

The current study focused on the relations between parent talk at the aquarium and children’s talk about science during and after informal learning. We examined how parent-child conversations at the aquarium linked to their at-home reminiscing conversations. As such, we observed and recorded the naturalistic interactions of parent-child dyads as they explored a live animal exhibit in an aquarium. We later collected a recorded conversation from the dyad in which they reminisced about the aquarium visit.

Transcripts of the conversations at the aquarium were analyzed for elements of parent elaborative talk, including *wh*-questions and elaborative statements, adapting approaches by Benjamin et al. (2010) and Jant et al. (2014). We extend prior work by also analyzing parent talk for closed-ended questions and repetitions (Reese et al., 1993; Peterson and McCabe, 1994), acknowledging that families use a diverse array of conversational styles during informal learning. Research generally reports no difference in parent-child reminiscing by parent education or income (Fivush et al., 2006). Differences in reminiscing by child gender have been mixed (Farrant, 2000; Newcombe and Reese, 2004), with some studies showing that parents are more elaborative with their daughters than sons (Reese et al., 1996) and others finding no differences (Laible, 2004; Melzi et al., 2011). During informal science interactions, however, parents explain more to boys than girls (Crowley et al., 2001b). For our first research aim, we explored the extent to which parents used elaborative talk with their children during informal learning at an aquarium and what, if any, differences occurred across family demographics.

Also, children’s talk at the aquarium and at home was coded for references to science processes, technology, math, and biological science content (henceforth: STMB), using a coding system developed for the aquarium (Kelly et al., 2020). For our second research aim, we investigated what type of parental talk at the aquarium predicted children’s science talk at the aquarium and at home. We expected that parents who used more elaborative talk at the aquarium would have children who used more STMB talk at the aquarium and at home (Benjamin et al., 2010; Jant et al., 2014). Lastly, reminiscing conversations were also investigated for the degree to which families reflected on valuing the aquarium experience, adapting work by Pagano et al. (2019). While some parents value museum visits more for recreational family experiences than for children’s learning (Letourneau et al., 2017), others value seeing their children’s learning during their visit (Luke et al., 2019). Our third research aim examined whether parental talk at the aquarium related to parent-child talk about valuing the aquarium visit at home. The research aims that investigate the relations of parent talk in the aquarium with child home STMB talk and dyad value talk were explored with pilot data from a subsample of families who recorded home conversations.

## Materials and methods

### Participants

The sample included 62 children (31 girls; *M*_age_ = 69.8 months, *SD* = 21.04) and 62 parents (35 mothers; *M*_age_ = 36.7 years, *SD* = 6.26) from a larger study of families^1^ who were recruited using convenience sampling at an aquarium in Southern California. One parent-child dyad was excluded because they primarily spoke in a language other than English during observations. Participants came from diverse racial/ethnic and socioeconomic backgrounds (see Table 1). Families were included if they had at least one child that was between three and eight years old and the child was accompanied by a legal guardian. Families received a small thank you gift for participating.

**TABLE 1:** Descriptive statistics for child, parent, and family demographic variables.

	Survey only *n* = 153	Aquarium observation^i^ *n* = 50	Home conversations^ii^ *n* = 25
Child age in months *M (SD)*	70.8 (22.6)	70.9 (20.4)	66.3 (19.7)
**Child gender**			
Girl	75 (0.49)	27 (0.54)	11 (0.44)
Boy	78 (0.51)	23 (0.46)	14 (0.56)
**Parent**			
Mother	82 (0.54)	29 (0.58)	14 (0.56)
Father	71 (0.46)	21 (0.42)	11 (0.44)
**Race/Ethnicity**			
Asian	21 (0.14)	6 (0.12)	4 (0.16)
Black	9 (0.06)	0 (0)	0 (0)
Latinx	39 (0.26)	16 (0.32)	4 (0.16)
Mixed-race	33 (0.22)	8 (0.16)	5 (0.20)
Native American	2 (0.01)	0 (0)	0 (0)
White	44 (0.29)	19 (0.38)	12 (0.48)
Other	4 (0.03)	1 (0.02)	0 (0)
**Family income**			
Less than $35K	22 (0.14)	4 (0.08)	3 (0.12)
35K–$75K	20 (0.13)	7 (0.14)	4 (0.16)
75K–$100K	24 (0.16)	9 (0.18)	3 (0.12)
Greater than $100K	80 (0.52)	27 (0.54)	15 (0.60)
**Parent education**			
Some college or less	53 (0.35)	19 (0.38)	4 (0.16)
Bachelor’s degree	50 (0.33)	14 (0.28)	6 (0.24)
Graduate degree	49 (0.32)	17 (0.34)	15 (0.60)

^*i*^One dyad was excluded from the aquarium observation group because they conducted their conversation primarily in a language other than English and, therefore, could not be included in analyses.

^*ii*^ The home observation subsample included 13 dyads from the aquarium observation group and 12 dyads from the survey only group.

### Procedure and measures

#### Aquarium

Participants were recruited at one of two live sea animal exhibits, which were comparable in length and location.

For the observation group (*n* = 50), researchers invited one child and one parent to participate at the exhibit entrance. If the family group included more than one eligible child, the parent(s) chose which child would participate. After informed consent, researchers gave parents a Bluetooth microphone to place on their clothing and children wore a backpack with a microphone on the strap. Researchers observed and audio recorded parent-child dyads while they explored the exhibit. At the end of exhibit, parents completed the study survey. For the survey group (*n* = 153)^2^, researchers recruited families at the exhibit exit. After informed consent, parents completed the study survey. The survey asked for child and parent gender and age, parent education, and family income.

#### Home follow-up

Twenty-five dyads were retained for the study follow-up including 13 from the observation group and 12 from survey group. To collect home conversations, a researcher contacted parents within two weeks and provided step-by-step instructions. Parents were instructed to record a home conversation with the target child using either a researcher-provided audio recorder or the voice memo app on their personal smartphones. Parents were asked to go to a quiet area with their child and “talk with your child about the day at the aquarium as you normally would.” Parents returned the recorders by mail in a prepaid envelope, or parents texted the voice memos to the researcher.

#### Parent-child conversations

Trained research assistants transcribed verbatim the recorded parent-child conversations using a version of the CHAT language transcription system (MacWhinney, 2000) and verified transcripts for accuracy. Parent and child language was parsed at the utterance level. The end of the utterance was determined by intonation and pauses or by coinciding with grammatically or meaningfully cohesive units.

### Coding

The principal investigator (second author) and trained research assistants coded transcripts of the aquarium and home conversations.

#### Parental talk

Using a coding system that bridges approaches in applied linguistics and developmental psychology, we first identified whether parent utterances were declarative statements or questions to capture the full range of parent utterances. Then using codes adapted from Reese et al. (1993) and Haden et al. (2014), we coded parent questions as either *wh*-questions, which were open-ended requests for new information relating to the aquarium, exhibit or sea animals (e.g., “*Where is the Bluestreak Cleaner?*”), or either/or questions, which were closed-ended requests that require a simple confirmation or negation of information (e.g., “*Do you like this jellyfish?*”) or choice from a finite set of options offered by the speaker (e.g., “*Is it a puffer fish or a clownfish?*”). We coded parent declarative statements that provided new information relating to the aquarium, exhibit, or sea animals as elaborative statements (e.g., “*Look they’re upside down*”). Requested or offered information that the parent asked or provided previously was coded as repetition (e.g., “What is it? *What is that?*”).

#### Child STMB talk

We measured children’s talk about science at the aquarium and at home using the STMB coding system developed for informal learning at the aquarium by Kelly et al. (2020). Child STMB talk included: science process - talk that problem-solved; characterized shape, color, or size; compared similarities or differences; and hypothesized (e.g., “What are they doing? They’re *exploring the ocean*.”); technology - mentions of technology in the exhibit (e.g., “What does *that button do?*”); mathematics – talk of numbers or geometric shapes and that quantified an amount (e.g., “What shape is its body? *A triangle.*”); and biological science - talk specific to biological, ecological, and life science contexts including life cycle, biological function or labeling using a scientific animal name (e.g., “Oh, they’re good at *camouflaging*.”).

#### Dyad value talk

We measured the extent to which parents and children revealed valuing their experiences at the aquarium using the value coding system by Pagano et al. (2019). Parent and child utterances coded for value talk included mentions of what the experience meant to the visitor, including emotional expressions (e.g., “*I like petting them*”), mentions of learning (e.g., “*What else did you learn about?*”), and evaluations of their experiences (e.g., “When he said the penguins are in a rush, *that was super funny*”).

#### Reliability

The second author and trained research assistants used 20% of the transcripts to establish reliability. Cohen’s kappa was *k* = 0.88 for parent talk, *k* = 0.77 for child STMB talk, and *k* = 0.76 for dyad value talk. Disagreements were resolved by consensus and included in the final dataset. Research assistants coded the remaining data.

## Results

### Preliminary analysis

Preliminary analyses focused on differences in outcome variables (child STMB talk and dyad value talk) to explore potential covariates. Table 2 shows bivariate correlations between covariate and criterion variables. Families’ visit length averaged 16.3 min in the exhibits (range = 2–32 min). Visit length correlated with all four parent talk variables, *p*s < 0.025, as well as child STMB talk at home and dyad value talk at home, *p*s < 0.05. Thus, visit length was included in analyses with criterion variables measured in the home conversations. The number of adults and children varied for each family. Number of children negatively correlated with parent *wh*-questions and either/or questions, *p*s ≤ 0.02. No significant correlations were found with the criterion variables, *p*s > 0.18. Child age, parent education, nor family income were correlated with any of the criterion variables, *p*s > 0.20. Separate analyses of variance (ANOVAs) revealed all but one relationship between the remaining background variables (child gender, adult gender, and race/ethnicity) and the criterion variables was non-significant, *p*s > 0.45. Children used more STMB talk at the aquarium with fathers (*M* = 16.67, *SD* = 12.46) than with mothers (*M* = 9.59, *SD* = 8.52), *F*(1, 48) = –2.38, *p* = 0.021. Thus, parent gender was included in analyses with child aquarium STMB talk.

**TABLE 2:** Bivariate correlations between covariates and outcomes variables.

	1	2	3	4	5	6	7	8	9
**Covariates**									
1. Child age	–								
2. Parent education	–0.15	–							
3. Family income	–0.04	0.51**	–						
4. Visit length	0.22	0.14	0.11	–					
5. Number of adults	0.27*	–0.10	0.03	0.14	–				
6. Number of children	0.49*	0.07	0.22	0.07	0.48**	–			
**Outcomes**									
7. Child aquarium STMB talk	0.06	0.18	–0.003	0.19	0.19	0.003	–		
8. Child home STMB talk	0.08	0.09	0.22	0.66*	0.08	–0.07	0.42	–	
9. Dyad value talk	0.27	0.01	0.21	0.64*	0.12	0.08	0.20	0.79**	–

*p < 0.05, **p < 0.01.

### Main analyses

#### Parent elaborative talk and family demographics during informal learning

**FIGURE 1:**
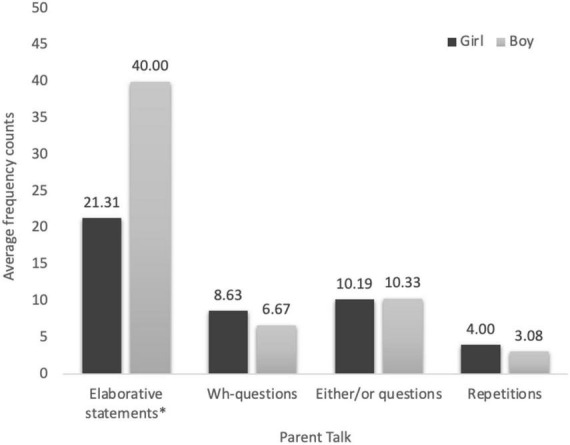
Average frequency of parent talk by child gender.

We first examined the descriptive statistics of the four parent talk variables. On average, parents used elaborative statements (*M* = 24.30, *SD* = 22.56) more than either/or questions (*M* = 9.34, *SD* = 7.61), which in turn were used more than *wh*-questions (*M* = 7.22, *SD* = 6.10) and repetitions (*M* = 3.86, *SD* = 5.56). Correlations and multivariate analysis of covariance (MANCOVA) examined relations between parent talk and parent, child, and family demographics (child age, gender, parent gender, education, and family income), with relevant covariates. Child age was negatively correlated with parent *wh-*questions [*r*(47) = –0.33, *p* = 0.023], either/or questions [*r*(47) = –0.37, *p* = 0.008], and repetitions [*r*(47) = –0.38, *p* = 0.007], controlling for visit length. Figure 1 shows the average frequency and standard errors for parent talk at the aquarium by child gender. MANCOVA revealed parents used more elaborative statements with boys (*M* = 40.00, *SD* = 35.50) than with girls (*M* = 21.31, *SD* = 14.40), *F*(1, 47) = 5.16, *MSE* = 353.53, partial η^2^ = 0.099, *p* = 0.027. As indicated in Figure 1, parents averaged more *wh*-questions and repetitions with girls than with boys, but the difference was not statistically significant.

#### Parent talk at the aquarium with child STMB talk and dyad value talk

Children averaged 12.56 (range: 0–47, *SD* = 10.83) instances of STMB talk in the aquarium and 13.04 (range 1–65: *SD* = 15.37) instances of STMB talk at home. Dyads averaged 14.75 (range: 1–42, *SD* = 11.51) instances of value talk at home. We conducted multiple linear regressions, using the stepwise method, to explore initial models, predicting separately child aquarium STMB talk, home STMB talk, and dyad value talk at home based on parent elaborative talk (i.e., *wh*-questions plus statements), either/or questions, and repetitions in the aquarium, with relevant covariates. Following a commonly used method (i.e., Reese et al., 1993; Laible, 2004; Leyva et al., 2020), we combined *wh*-questions and elaborative statements creating a single parent elaborative talk variable to simplify the models. We did so because the two variables highly correlate and both theoretically and empirically function together to create a rich conversational context (Fivush et al., 2006).

##### STMB talk at the aquarium

The regression for child STMB talk at the aquarium yielded *R*^2^ = 0.24, *F* (4,45) = 3.58, *p* < 0.013. The top of Table 3 displays the significant predictor variables. Consistent with our hypothesis, parental elaborative talk was significantly positively associated with child aquarium STMB talk over and above visit length, child age and parent gender, with an average increase of.41 instances of child aquarium STMB talk for every 1-unit increase in parental elaborative talk.

**TABLE 3:** Multiple linear regression models predicting child STMB talk at the aquarium (top), child STMB talk at home (middle), and dyad value talk at home (bottom) from parent talk in the aquarium with covariates.

Variables	*B*	*SE B*	b
**Aquarium STMB talk**			
**Covariates**			
Child age	0.04	0.08	0.07
Parent gender	5.03	2.98	0.23
Visit length	–0.10	0.24	–0.06
Parent talk			
Elaborative talk	0.17	0.07	0.41*
**Home STMB talk**			
**Covariates**			
Child age	0.19	0.28	0.16
Visit length	1.19	0.70	0.44
Parent talk			
Elaborative talk	–0.32	0.26	–0.49
Either/or questions	2.70	1.17	2.31^
Repetitions	–2.14	0.94	–2.78^
**Value talk**			
**Covariates**			
Child age	0.25	0.24	0.30
Visit length	0.69	0.54	0.37
**Parent talk**			
Elaborative talk	–0.11	0.20	–0.24
Either/or questions	1.54	90	0.91
Repetitions	–1.13	0.73	–0.45

^p < 0.063; *p < 0.05.

##### STMB talk at home

The regression for child STMB talk at home yielded *R*^2^ = 0.75, *F* (5,11) = 3.58, *p* = 0.08. The middle of Table 3 displays the significant predictor variables. Contrary to our expectations, parental elaboration was not significantly associated with child home STMB talk. Instead, parent either/or questions were marginally positively associated with child home STMB talk, with an average increase of 2.31 instances of child home STMB talk for every 1-unit increase in parent either/or questions at the aquarium. Also, parent repetitions were marginally negatively associated with child home STMB talk, with an average decrease of 2.87 instances of child home STMB talk for every 1-unit increase in parent repetitions.

##### Value talk at home

The regression for dyad value talk yielded *R*^2^ = 0.69, *F* (5,11) = 2.70, *p* = 0.13. The bottom of Table 3 displays the model results. Parent talk variables were not significantly associated with value talk, when controlling for time in the aquarium. Only time in the exhibit significantly predicted value talk.

## Discussion

This study explored whether and how parent-child conversations bridge children’s science learning across informal learning ecologies. Parents tended to use more elaborative statements compared to other talk types in the aquarium. Yet, the different types of questions and statements that parents used with their children in the aquarium differentially related to their children’s science talk in the aquarium and while reminiscing at home.

Children whose parents used more elaborative talk during informal learning talked more about science in the aquarium. Social interactions, including family conversations, guide children to attend to content and processes that are highly valued within their communities (Wood et al., 1976). Parents who asked for and provided new information during informal learning signal to their children what scientific content and processes are important to attend to and thus talk about. Our finding that naturally occurring elaborative talk in everyday family conversations provided children with opportunities to engage in science extends Jant et al.’s (2014) study, which cued parents to ask elaborative questions at a museum. Children’s learning can occur in everyday conversations (Falk and Dierking, 2000; Hirsch-Pasek et al., 2015), which, as our results suggest, promotes their developing scientific thinking and knowledge.

Additionally, parents used more elaborative statements with boys than with girls in the aquarium. This finding is consistent with studies on parent scientific explanations (Crowley, 2000; Crowley et al., 2001b). In our study, this disparity may have been mitigated by parents’ use of slightly more *wh*-questions and repetitions with girls, as there were no gender differences in child STMB talk. Nevertheless, this finding is a reminder for ILE educators and designers of the pernicious presence of gendered behaviors in family informal science interactions, which can have long-term ramifications for children’s scientific literacy (Tenenbaum et al., 2005).

Contrary to our expectations, parental elaborative talk in the aquarium was not related to child science talk while reminiscing at home. Instead, it appears that children whose parents used more either/or questions may talk more about science while reminiscing about the aquarium. These results must be taken with caution due to the reduction of our retained sample for the home conversations. Nevertheless, these preliminary findings add dimension to a body of research that has often focused on the role of parent *wh*-questions or elaborative statements in children’s informal science learning (e.g., Boland et al., 2003; Jant et al., 2014). Because families from different backgrounds bring to informal learning their *FoK* (Moll et al., 1992) and ways of talking with each other (Heath, 1983; Carmiol et al., 2019), our findings highlight the importance of investigating the full range of conversational styles in ILE.

Often-overlooked types of parent talk likely provide meaningful ways for families to engage in science and lead to positive child learning outcomes. Thus, this study broadens past informal learning work by considering all types of talk beyond privileged and formal ways of talking about science (i.e., causal talk and science explanations) (Callanan and Oakes, 1992; Joy et al., 2021). From a *FoK* perspective, all family conversation styles should be considered and leveraged across learning ecologies for children’s science learning, highlighting that knowledge and skills are acquired through everyday experiences that are historically and culturally unique (Moll et al., 1992). Our study expands the boundaries of what we define as science engagement in ILE and emphasizes that participation, conversations, and engagement in these spaces is what counts as science (Callanan et al., 2013).

Finally, parent talk in the aquarium was not related to the degree to which dyads reflected on the value of the aquarium visit. Only the amount of time that families spent in the exhibit was predicted value talk at home. Although the direction of the relationship cannot be determined due to the correlational nature of the study, this finding is consistent with theories of learning with adults (Astin, 1984) and studies of effective educational practice (Chickering and Gamson, 1987) that time spent on task is a powerful predictor of educational effectiveness. Debate about the role of time spent in museums is longstanding (e.g., Serrell, 1997; Shettel, 1997). Nonetheless, that the time a family spent in the aquarium predicted the extent to which they later revealed valuing the experience has meaningful implications for exhibit design. For example, exhibits that are intended to foster values, such as conservationism or multicultural awareness, should have a longer hold time for lasting impact.

### Limitations

Certain study features limit our conclusions. We focused only on *how* parents talked with their children during informal learning. Future research examining the content of parent talk could reveal the relative contributions of quality versus content of informal learning conversations to understand better the signals that parents send to their children about what is valued as science. Also, we experienced sample attrition in the follow-up, despite our best efforts to make the follow-up easy and free for families. Not only did the small sample likely reduce statistical power in our regression analyses, it also limits the extent to which we can generalize the findings of links between parent talk in the aquarium and talk at home. The results from the pilot home data are preliminary but show promise for future studies with larger samples. Future studies should implement new video conferencing technologies into procedures, which may streamline the recording and collection of home conversations. Although we cannot draw conclusions about causality, we believe the naturalistic study design is a strength for its ecological validity.

### Conclusions

This study provides new information about the kinds of parent-child interactions that may promote children’s talk about science content as well science processes during informal learning and across learning ecologies. The results suggest that ILE educators can model a conversational style that includes abundant either/or questions and *wh*-questions, acknowledging and leveraging families’ existing strengths to support learning in aquariums and other ILEs. Young learners see connections to science across the everyday settings in which they participate, including school, home, and community (Zimmerman and Bell, 2014). Continuing to identify ways to bridge children’s learning ecologies can enhance theoretical understandings of how science learning in one setting can promote learning across other learning contexts. Finding ways to create “seamless ecologies” (Meyers et al., 2013) can empower families to use their strengths across familiar and new settings to promote science learning.

## Data availability statement

The raw data supporting the conclusions of this article will be made available by the authors, without undue reservation.

## Ethics statement

The studies involving human participants were reviewed and approved by Institutional Review Board’s Committee at California State University Long Beach. Written informed consent to participate in this study was provided by the participants’ legal guardian/next of kin.

## Author contributions

KK conceptualized and designed the study. GO, KK, BH, and CM contributed to data collection. GO, KK, and LM contributed to data coding and database organization. KK performed the statistical analysis. GO wrote the first draft of the “Materials and methods” section, and KK wrote the first draft of the “Results” section. GO and KK contributed equally to the “Introduction” and “Discussion” sections. SN and KA contributed to the literature review. GO and KK revised the manuscript, read, and approved the submitted version. All authors contributed to the article and approved the submitted version.

## Footnotes

1 The larger study sample included *N* = 204 parent-child dyads, which consists of dyads who completed only the study survey (*N* = 153) and those who completed an additional observation (*N* = 51).

2 Data for families who agreed to the survey only procedures but did not complete the follow-up home observation (*n* = 141) were not included in the current study.
